# Adolescents’ affective and neural responses to parental praise and criticism

**DOI:** 10.1016/j.dcn.2022.101099

**Published:** 2022-03-15

**Authors:** Lisanne A.E.M. van Houtum, Geert-Jan Will, Mirjam C.M. Wever, Loes H.C. Janssen, Charlotte C. van Schie, Marieke S. Tollenaar, Bernet M. Elzinga

**Affiliations:** a Department of Clinical Psychology, Institute of Psychology, Leiden University, Leiden, The Netherlands; b Leiden Institute for Brain and Cognition (LIBC), Leiden, The Netherlands; c Illawarra Health and Medical Research Institute and School of Psychology, University of Wollongong, Wollongong, Australia

**Keywords:** Social feedback, Parental criticism, Parental praise, FMRI, Adolescence, Self-views

## Abstract

Social feedback from parents has a profound impact on the development of a child’s self-concept. Yet, little is known about adolescents’ affective and neural responses to parental social feedback, such as criticism or praise. Adolescents (*n* = 63) received standardized social feedback supposedly provided by their mother or father in the form of appraisals about their personality (e.g., ‘respectful’, ‘lazy’) during fMRI scanning. After each feedback word, adolescents reported their mood. Additionally, adolescents had rated whether feedback words matched their self-views on an earlier occasion. In line with preregistered hypotheses, negative parental feedback worsened adolescents’ mood, which was exacerbated when feedback did not match adolescents’ self-views. Negative feedback was associated with increased activity in the neural ‘saliency network’, including anterior insula, anterior cingulate cortex and dorsomedial prefrontal cortex. Positive feedback improved mood and increased activity in brain regions supporting social cognition, including temporoparietal junction, posterior superior temporal sulcus, and precuneus. A more positive *general* self-view and perceived parental warmth were associated with elevated mood, independent of feedback valence, but did not impact neural responses. Taken together, these results enhance our understanding of adolescents’ neural circuitry involved in the processing of parental praise and criticism, and the impact of parental feedback on well-being.

## 1 Introduction

During adolescence, interpersonal sensitivity is typically exacerbated, which is mirrored in neurobiological changes in the adolescent brain and reorganizations in the social context, such as spending more time with friends versus family (Nelson et al., 2005, Steinberg and Silk, 2002). Moreover, social feedback becomes increasingly important for the formation and development of adolescents’ self-views (Becht et al., 2017, Chen et al., 2006). Although adolescents usually strive for autonomy to individuate themselves from their parents (Steinberg and Silk, 2002), parents remain a vital source of feedback (Grotevant and Cooper, 1985, McLean, 2005, Welborn et al., 2016). Both parental negative and positive feedback can form adolescents’ self-views in crucial ways (Brummelman and Thomaes, 2017, Harter, 2015, Jacquez et al., 2004), even far into adulthood (Koepke and Denissen, 2012). Yet, little is known about adolescents’ neural reactivity to parental feedback, and how adolescents’ own self-views shape the way they deal with this feedback. Moreover, it is unknown how the processing of parental feedback is shaped by the warmth and criticism adolescents receive from their parents in daily life. Therefore, this study examines how parental feedback in the form of criticism and praise impacts adolescents’ mood and neural responses, and whether this depends on the consistency of the feedback with adolescents’ existing views of themselves and/or on their perceptions of daily parental warmth and criticism.

Despite parents’ benevolent intentions, parental criticism is generally experienced as a social threat, associated with negative emotions (Harris and Howard, 1984). Indeed, persistent parental criticism may instill a chronic negative self-image in adolescents, which in turn makes them vulnerable to developing mental health issues, such as depression, even into adulthood (Harris and Howard, 1984, Jacquez et al., 2004, Robertson and Simons, 1989, Sheeber et al., 2001). On a neural level, receiving negative social feedback in general (i.e., from *un*known others) has been consistently associated with increased activation in brain regions implicated in affect and saliency, such as anterior cingulate cortex (ACC) and anterior insula (AI; Cacioppo et al., 2013; Eisenberger et al., 2011; Feng et al., 2021; Fritz et al., 2020; Kawamichi et al., 2018; Muscatell et al., 2016; Rotge et al., 2015; Schindler et al., 2019; van Schie et al., 2018; Will et al., 2016).

Parental praise, on the other hand, is usually experienced as rewarding and induces positive emotions and increases self-esteem, self-efficacy, and motivation (Brummelman and Thomaes, 2017, Jacquez et al., 2004, Owen et al., 2012). Positive social feedback in general reliably activates ventral striatum (VS) and ventromedial prefrontal cortex (vmPFC; Davey et al., 2010; Feng et al., 2021; Gunther Moor et al., 2010; Izuma et al., 2008; Kawamichi et al., 2018; Korn et al., 2012; Morelli et al., 2015; Muscatell et al., 2016; Schindler et al., 2019; Will et al., 2020, 2017). Processing social feedback, independent of their valence, also elicits socio-cognitive processes, such as mentalizing (i.e. understanding others’ mental states), perspective-taking and self-referential thinking. These socio-cognitive processes help to interpret the personal relevance of feedback, relate it to the messenger, and integrate it with own self-views (Silk et al., 2017). A number of brain regions is involved in these processes, including dorsomedial PFC (dmPFC), temporoparietal junction (TPJ), posterior superior temporal sulcus (pSTS) and posterior cingulate cortex (PCC)/precuneus (Kawamichi et al., 2018, Molenberghs et al., 2016, Muscatell et al., 2016, Pfeifer et al., 2009, Schurz et al., 2014, van Houtum et al., 2021, Van Overwalle, 2009, van Schie et al., 2018).

So far, in most fMRI studies participants receive social feedback from strangers. *Parental* feedback may involve other processes and co-activate other neural networks, for example involved in attachment and autobiographical memory processing (Silk et al., 2017). To our knowledge, only one study has been published about neural responses to parental feedback in healthy adolescents receiving auditory criticism from their mother. Here, criticism (vs. neutral comments, e.g. about the weather) was associated with *decreased* activity in ACC, TPJ and PCC/precuneus (Lee et al., 2015) rather than increased activation that is typically found in these areas after negative feedback. Given the significance of parental feedback in fostering either a positive or negative self-image (Brummelman and Thomaes, 2017, Harris and Howard, 1984, Owen et al., 2012), it is important to examine how parental feedback, both positive and negative, impacts on adolescents’ affective and neural responses.

Several factors – both intra- and interpersonal – may determine to what extent social feedback resonates and affects a person’s wellbeing. First, feedback that is consistent with own self-views is processed more easily and experienced as more pleasant, as it confirms existing beliefs (Stinson et al., 2010, van Houtum et al., 2021, van Schie et al., 2018). Moreover, in adults applicable (vs. inapplicable) feedback from a confederate yielded increased precuneus activation (van Schie et al., 2018). When feedback is *inconsistent* with self-views, individuals are usually more reluctant to accept it and report exacerbated negative effects on mood (Sedikides and Gregg, 2008, van Houtum et al., 2021, van Schie et al., 2018). Work on self-evaluations shows that evaluating negative personality characteristics that are considered as applicable engage vmPFC and pregenual ACC (pgACC) to a greater extent than inapplicable negative characteristics, while the opposite was found for positive characteristics (Barendse et al., 2020, Cosme et al., 2019). vmPFC has been linked to signaling personal relevance, showing increased activation to self-relevant stimuli independent of valence (D'Argembeau, 2013). As it is unclear whether self-relevance is also a key factor for adolescents when processing parental feedback, we aimed to study whether and how applicability of parental feedback impacts adolescents’ affective and neural responses.

Secondly, individuals tend to hold (biased) favorable views of themselves (Murray et al., 1996, Sedikides and Gregg, 2008, Sharot and Garrett, 2016, Taylor and Brown, 1988). Having an overall positive, stable self-view has been related to a variety of positive outcomes, such as psychological well-being, health development, social functioning and academic achievements (Harter, 2015, Sharot and Garrett, 2016, Taylor and Brown, 1988). Based on the notion of consistency, one might expect that adolescents who generally view themselves more positively show amplified mood responses to parental praise, as this confirms their self-views to a larger extent (Alicke et al., 2020). Along a similar line of reasoning, one might expect that parental criticism has a stronger negative impact in adolescents who generally view themselves more positively (since it is more likely that the negative feedback is inconsistent with their self-views). However, this effect may be canceled out by the fact that people with higher self-esteem generally seem to be less vulnerable to criticism (Baldwin, 2006, Vandellen et al., 2011). Taking these two considerations into account, we do not expect to find a strong impact of general self-view on mood in response to negative feedback. As it remains to be elucidated whether adolescents’ *general* self-view influences neural activity in response to social evaluations from parents, this will be another study aim.

Lastly, an *inter*personal factor that might impact adolescents’ responses to parental feedback is exposure to parental warmth and criticism in daily life. According to the parental acceptance-rejection theory, adolescents receiving less parental warmth may develop a weaker sense of safety and self-worth, and hence are more likely to perceive threat in interpersonal contexts (Butterfield et al., 2020, Rohner et al., 2005). This might imply that these adolescents perceive parental criticism as more threatening, whereas they may show diminished responses to parental praise. On a neural level, Lee et al. (2015) found that less parental warmth correlated with decreased TPJ and precuneus activation in response to maternal criticism. Other research found that adults perceiving their mother as more critical showed decreased dorsolateral PFC and increased amygdala activation in response to maternal criticism (Hooley et al., 2012). However, in these studies parental warmth and criticism were not investigated on a *daily* basis*.* Measuring parenting behaviors through ecological momentary assessments (EMA; Stone and Shiffman, 1994) provides a unique opportunity to capture (perceived) behaviors in everyday circumstances over an extended period of time (in our case: two weeks). Combining daily life assessments with fMRI can potentially uncover relevant and ecologically valid brain–behavior relationships related to social processes and feedback (Powers et al., 2016).

In sum, in this study we aim to elucidate adolescents’ affective and neural responses to parental praise and criticism, assessed with a task including positive, negative and intermediate social feedback. To ensure both ecological *and* internal validity, we use (fake) standardized parental feedback, which is identical for every adolescent (see also van Houtum et al., 2021). Secondly, we aim to examine if (in)consistency of feedback with adolescents’ own self-views and their *general* self-view impacts adolescents’ affective and neural responses to such feedback. Third, we aim to explore whether individual differences in adolescents’ perceived parental warmth and criticism in daily life moderate adolescents’ affective and neural responses. In contrast to existing fMRI research on parental feedback (that only included feedback from mothers), roughly 50% of our participants receives feedback from their father, allowing for more generalizable conclusions.

All study measures and hypotheses were preregistered at Open Science Framework prior to data analyses (https://osf.io/5nj76/). We hypothesize that parental positive feedback (vs. intermediate/negative feedback) increases mood, while negative feedback (vs. intermediate/positive feedback) decreases mood (Jacquez et al., 2004, van Schie et al., 2018). In terms of brain responses, we expect that positive feedback increases activity in VS and vmPFC (Davey et al., 2010, Feng et al., 2021, Gunther Moor et al., 2010, Izuma et al., 2008, Kawamichi et al., 2018, Korn et al., 2012, Morelli et al., 2015, Muscatell et al., 2016, Schindler et al., 2019, Will et al., 2020, Will et al., 2017), whereas negative feedback increases activity in ACC and AI (Cacioppo et al., 2013, Eisenberger et al., 2011, Feng et al., 2021, Fritz et al., 2020, Kawamichi et al., 2018, Muscatell et al., 2016, Rotge et al., 2015, Schindler et al., 2019, van Schie et al., 2018, Will et al., 2016). Given previous mixed findings, we explore whether activation in brain regions important for socio-cognitive processing (e.g. dmPFC, PCC/precuneus, TPJ, pSTS) decreases (Lee et al., 2015) or increases (van Schie et al., 2018) in response to parental feedback as a function of feedback valence. We furthermore expect that inapplicable feedback decreases mood, particularly inapplicable negative feedback (van Schie et al., 2018). We hypothesize that adolescents with a more positive *general* self-view report overall higher mood (Harter, 2015) and amplified mood responses to positive feedback, whereas negative feedback impacts all adolescents similarly (Alicke et al., 2020, Vandellen et al., 2011). We expect that adolescents reporting less parental warmth in daily life exhibit larger decreases in mood in response to negative parental feedback, and smaller increases in mood to positive feedback compared to those reporting higher levels of parental warmth (Rohner et al., 2005). Lastly, we explore how individual differences in self-views and perceived parenting in daily life modulate neural responses to parental feedback.

## 2 Methods

### 2.1 Participants

Adolescents and one or both of their parents participated in a Dutch multi-method two-generation study called RE-PAIR (*‘Relations and Emotions in Parent-Adolescent Interaction Research’*), investigating the bidirectional interplay between parent-adolescent social interactions and mood in adolescents with and without major depressive disorder (MDD) or dysthymia. Analyses for the current paper were restricted to the healthy adolescents of the RE-PAIR study. Inclusion criteria for healthy adolescents were: aged between 11 and 17 years at the time of the first lab session, having started secondary school, living with one or both parents, no lifetime diagnosis of MDD or dysthymia or any other psychiatric diagnosis in the two years preceding study participation (assessed using Kiddie-Schedule for Affective Disorders and Schizophrenia–Present and Lifetime Version (K-SADS-PL; Kaufman et al., 1996)), and good command of the Dutch language. For the fMRI part of the study (i.e. scanning session), MRI-incompatibility (i.e., implanted medical devices, non-removable metal in the body, pregnancy, claustrophobia) was specified as exclusion criterion.

In total, 63 adolescents took part in the scanning session. Two adolescents were excluded due to scanner artefacts; one due to excessive head motion (as preregistered, see https://osf.io/5nj76/). Although not preregistered as exclusion criterion, one adolescent was excluded because of depression severity scores in the clinical range as reported on the Patient Health Questionnaire (PHQ; Kroenke and Spitzer, 2002, Kroenke et al., 2001) (i.e. PHQ-score of 18). This was also reflected in the affective data of the feedback task (i.e. >3 SD below the mean mood after positive parental feedback). This resulted in a final sample of 59 adolescents (see Table 1 for demographics). Four adolescents reported medication use for physical ailments at the day of scanning (hay fever/allergy medication (H_1_-antagonist): *n* = 2; asthma inhaler (long-acting-β_2_-agonist): *n* = 1; anti-inflammatory pain reliever (NSAID): *n* = 1).

**Table 1:** Participants’ demographics and descriptive statistics (*n* = 59).

Variables	Mean (SD)/n (%)	Range
**Age adolescent (years)**	16.2 (1.21)	12.6–18.2
**Sex adolescent, n male (%)**	20 (33.9%)	–
**Sex parent, n male (%)**	27 (45.8%)	–
**Current educational level, n (%)**		
Lower vocational (VMBO)	7 (11.9%)	–
Higher vocational (HAVO)	19 (32.2%)	–
Pre-university (VWO)	26 (44.1%)	–
Secondary vocational (MBO)	5 (8.47%)	–
Higher professional (HBO)	2 (3.39%)	–
**Handedness (EHI-score)**	71.0 (52.9)	-100–100
Right-handed, n (%)	54 (91.5%)	
**Pubertal development (PDS-score)**	3.25 (0.63)	1–4
**Depressive symptoms (PHQ-score)**	4.36 (2.52)	0–12
**Parent-child bonding (PBI-score)**^**1**^		
Care	30.8 (5.13)	14–36
Overprotection	8.69 (4.62)	0–22
**General self-view**	0.98 (0.45)	0.14–2.14
**Daily Perceived Warmth EMA**^**2**^	5.86 (0.83)	3.71–7.00
**Daily Perceived Criticism EMA**^**2**^	1.90 (0.97)	1.00–4.70

Notes: ^1^n = 58, as PBI data of one adolescent boy was missing. ^2^n = 57, as two adolescent girls were not included due to insufficient data about perceived parental warmth and criticism in daily life. Abbreviations: EMA = Ecological momentary assessments; EHI = Edinburgh Handedness Inventory (Oldfield, 1971); HAVO = Senior general secondary education; HBO = Higher professional education; MBO = Secondary vocational education; PBI = Parental Bonding Instrument; PDS = Pubertal Development Scale (Petersen et al., 1988); PHQ = Patient Health Questionnaire (Kroenke and Spitzer, 2002); VMBO = Pre-vocational secondary education; VWO = Pre-university education.

The study was approved by the Medical Ethics Review Committee (METC) of Leiden University Medical Centre (LUMC) in Leiden, the Netherlands (reference: P17.241; protocol: NL62502.058.17) and conducted in accordance with the Dutch Medical Research Involving Human Subjects Act (WMO) and Declaration of Helsinki.

### 2.2 Procedure

After initial phone screening, families filled out several online questionnaires, such as the Parental Bonding Instrument (PBI; Parker et al., 1979) to assess parent-child bonding, and were invited for a lab session. During this session, adolescents and their parents provided written informed consent. Next, they performed several tasks and questionnaires, including questions about personality characteristics of the adolescent. After the lab session, families completed EMA for 14 consecutive days on their smartphones using an app called Ethica (see https://ethicadata.com/product). Adolescents reported daily on perceived parental warmth and criticism (for more detailed information, see Janssen et al., 2020).

Adolescents and one of their parents were invited for an MRI-scanning session (scheduled ≥ one week after the lab session: *M* = 7.36 weeks, *SD* = 6.30, *range:* 1.86–37.86; families generally started with EMA the first Monday following their first lab session; except in case of holidays and/or adolescents’ exam weeks, then EMA started the first Monday after holidays/exams (length of interval between end of EMA and MRI session: *M* = 3.11 weeks, *SD* = 7.04, *range:* −13.29 to 31.00)). Participants provided written informed consent again, were accustomed to the scanning environment by means of a mock-scanner and received detailed task instructions. Adolescents performed four tasks in the MRI-scanner (i.e., an eye-contact task, the parental social feedback task (as described here), a peer evaluation task, and an autobiographical memory task). Before and after each task, adolescents filled out visual analog scales (VAS) to assess their current level of self-esteem, sadness, relaxation and irritation. We counterbalanced the order of the parental social feedback task and peer evaluation task to control for carry-over effects. No between-group differences were found with regard to VAS-scores before or after the parental social feedback task (all *p*-values >0.312).

Upon completion of scanning, adolescents filled out several questionnaires, including the PHQ (Kroenke and Spitzer, 2002) and Edinburgh Handedness Inventory (EHI; Oldfield, 1971). Finally, adolescents were subjected to a manipulation check interview to assess whether they believed the cover story that feedback was provided by their parent. No adolescent disbelieved our cover story (see Supplementary Material 1). Hereafter, adolescents were debriefed about the study purpose and reasons for preprogramming the parental feedback. Adolescents were first debriefed alone to ensure they understood that feedback was preprogrammed and not based on their parent’s appraisal of their personality. Subsequently, we informed parents that we told their child that they received fake feedback and that we debriefed the child about this. Additionally, families received a letter explaining the experimental set-up and were asked whether they would like to be contacted later to evaluate their experiences (contacted families: *n* = 7). The task was well-received by families, and all families were positive about their study participation. Adolescents received €20 and their parents €30 for the scanning session plus compensation for travel expenses.

### 2.3 Materials

#### 2.3.1 Parental social feedback task

The parental social feedback task was based on a social feedback task previously developed in our lab, initially to investigate the neural correlates of social feedback from a stranger (van Schie et al., 2018). In the current, modified version, adolescents received social feedback (i.e., words describing personality characteristics) supposedly given by their *parent*. During the first lab session, adolescents rated 49 feedback words in terms of valence (*‘What do you think of this personality characteristic?’*) on a scale of −4 (*‘very negative’*) to 0 (*‘neutral’*) to 4 (*‘very positive’*) and in terms of applicability to the self (*‘To what extent does this personality characteristic apply to you?’*) on a scale of 1 (*‘not at all’*) to 5 (*‘very much’*). If the meaning of a feedback word was unclear, adolescents could answer the questions with a question mark. These feedback words were discarded from analyses on a person-based level (excluded words: *n* = 16 (0.6%) distributed across 12 participants: 1 word: *n* = 9; 2 words: *n* = 2; 3 words: *n* = 1).

Prior to performing the task in the scanner, adolescents were informed that their parent (the one present during the scanning session) was asked to select both positive and negative personality characteristics from a list that they deemed most descriptive of their child. In reality, each adolescent received the same preprogrammed feedback, split in three predetermined valence categories: 15 positive (e.g. *‘Sweet’*), 15 intermediate (e.g. *‘Nervous’*), and 15 negative words (e.g. *‘Unreliable’*; see van Houtum et al., 2021). These feedback words were presented in a semi-randomized fashion, such that consecutive feedback words were not of similar valence. The task always started and ended with two positive feedback filler words, which were excluded from analyses.

**Fig. 1:**
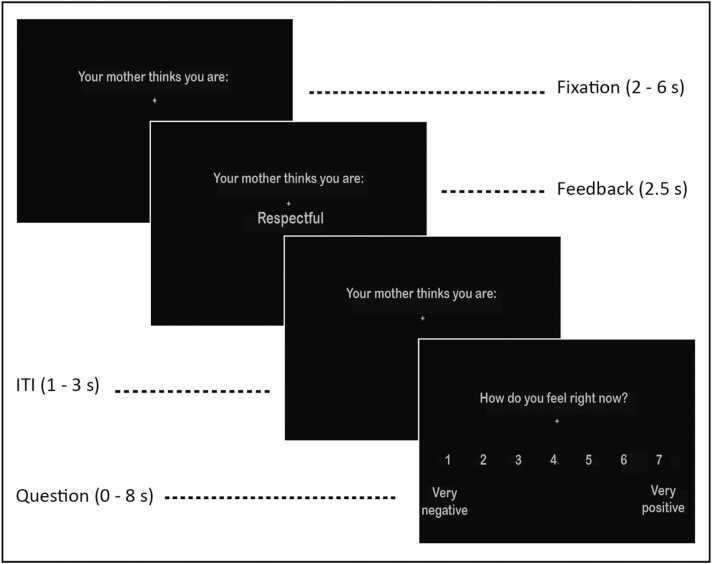
Trial structure of parental social feedback task. Dependent on the participating parent, *‘mother’* was replaced by *‘father’*.

Fig. 1 presents the trial structure of the parental social feedback task. Each trial started with a jittered fixation cross with a uniformly distributed duration varying between 2000 and 6000 ms (*M* = 4000 ms). The sentence *‘Your mother/father thinks you are:’* was shown on the screen during each trial. Next, a feedback word was displayed on the screen for 2500 ms, with a jittered inter-trial-interval fixation cross varying between 1000 and 3000 ms (*M* = 2000 ms). Following each feedback word, adolescents rated their current mood (*‘How do you feel right now?’*) on a scale ranging from 1 (*‘very negative’*) to 7 (*‘very positive’*) using MR-compatible button boxes. Participants used their left index- and middle finger to move from left to right on the scale and their right index-finger to confirm their responses. The mood question was self-paced and lasted for a maximum of 8000 ms (see Fig. 1). If adolescents failed to respond within the timeframe, the message *‘Too late’* was displayed for 1000 ms, and the trial was excluded from analyses (excluded trials: *n* = 4; 0.15%).

The task was programmed in E-Prime 2.0 (Psychological Software Tools, Pittsburgh, PA) and presented on a 32-inch BOLD-screen (Cambridge Research Systems, Cambridge, UK) placed at the end of the scanner bore, which participants could see via a mirror attached to the head coil.

#### 2.3.2 Adolescents’ general self-view

As preregistered, we calculated adolescents’ general tendency to view themselves positively by multiplying their applicability ratings of the feedback words with z-scored valence ratings of these words. We averaged these applicability*z-scored valence values over all feedback words per participant to create a general self-view score (van Houtum et al., 2021), see Table 1. A higher score indicated an overall more positive view of the self (i.e., many positive feedback words were rated as applicable, and many negative feedback words as inapplicable).

#### 2.3.3 Perceived parental warmth and criticism in daily life

During the two EMA-weeks – in the final questionnaire of each day – adolescents were asked to indicate with which parent(s) they interacted during that day, and if so, to rate each parent’s warmth (*‘Throughout the day, how warm/loving was your parent towards you?’*) and criticism (*‘Throughout the day, how critical was your parent towards you?’*) on a 7-point Likert scale ranging from 1 (*‘not at all’*) to 7 (*‘very’*). The ratings concerning the parent who ostensibly gave the feedback during our task were averaged over 14 days to create a perceived warmth and a criticism score (*n* days reported: *M* = 11.26, *SD* = 2.59, *range:* 6–14). Two adolescent girls were excluded from analyses about perceived parental warmth and criticism, due to a low completion rate (<25%; Sequeira et al., 2021).

#### 2.3.4 MRI data acquisition

MRI scans were acquired using a Philips Achieva dStream 3.0-Tesla scanner (Philips Medical Systems, Best, NL) equipped with a SENSE-32 whole-head coil. Head motion was restricted using foam inserts. First, we acquired a structural 3D T1-FFE scan (TR: 7.9 ms, TE: 3.5 ms, flip angle: 8°; 155 transverse slices; FOV: 250 ×195.83 ×170.5 mm; voxel size: 1.10 mm^3^; duration: 4:11 min). Next, fMRI images were collected with T2 * -weighted echo-planar imaging (EPI) sequence (TR: 2.2 s, TE: 30 ms, flip angle: 80°; 38 transverse slices (anterior-to-posterior); FOV: 220 × 220 × 114.68 mm; voxel size: 2.75 mm^3^). Number of volumes per participant varied due to self-paced questions (*M* = 237.8, *SD* = 10.5, *range:* 221–273). After obtaining functional scans, we collected field maps for correction of distortion in the EPIs (TR: 200 ms, TE: 3.2 ms; maximum: 58 slices (optimum: 29 slices); voxel size: 2.75 mm^3^).

### 2.4 Data pre-processing and analysis

#### 2.4.1 Behavioral data analysis

Behavioral data were analyzed using R-4.0.4 (R Core Team, 2013). We used lme4 for multilevel analyses (Bates et al., 2015) and ggplot2 for figures (Wickham, 2016). We analyzed how mood varied as a function of predetermined feedback valence using multilevel models (Hox et al., 2017), with intermediate feedback as reference category to which effects of positive and negative feedback were compared. Feedback valence categories were specified on the first level; adolescents’ mood after each feedback word was included as outcome:

$${\mathrm{Mood}}_{\mathrm{ij}}=\gamma_{00}+\gamma_{10}{\left(\mathrm{Negative}\right)}_{\mathrm{ij}}+\gamma_{20}{\left(\mathrm{Positive}\right)}_{\mathrm{ij}}+\upsilon_{0j}+\upsilon_{1j}{\left(\mathrm{Negative}\right)}_{\mathrm{ij}}+\upsilon_{2j}{\left(\mathrm{Positive}\right)}_{\mathrm{ij}}+\;\varepsilon_{\mathrm{ij}}$$

All continuous variables were standardized (i.e., subtracted by its mean and divided by its standard deviation) before estimation and consequently, the reported coefficients are standardized coefficients. All examined models include random effects for feedback valence. χ2-tests were used to test for significance of effects. To estimate effect sizes, we reported standardized coefficients and Cohen’s *f*^2^ (i.e., variance explained for the overall model as compared to the null model) (Cohen, 1992, Lorah, 2018).

To test if (in)consistency of feedback words with adolescents’ self-views has an effect on adolescents’ mood, self-rated applicability ratings were added on the first level to the model described above. The examined model includes random effects for both feedback valence and applicability:

$${\mathrm{Mood}}_{\mathrm{ij}}=\gamma_{00}+\gamma_{10}{\left(\mathrm{Negative}\right)}_{\mathrm{ij}}+\gamma_{20}{\left(\mathrm{Positive}\right)}_{\mathrm{ij}}+\gamma_{30}{\left(\mathrm{Applicability}\right)}_{\mathrm{ij}}+\gamma_{40}{\left(\mathrm{Negative}*\mathrm{Applicability}\right)}_{\mathrm{ij}}+\gamma_{50}{\left(\mathrm{Positive}*\mathrm{Applicability}\right)}_{\mathrm{ij}}+\upsilon_{0j}+\upsilon_{1j}{\left(\mathrm{Negative}\right)}_{\mathrm{ij}}+\upsilon_{2j}{\left(\mathrm{Positive}\right)}_{\mathrm{ij}}+\upsilon_{3j}{\left(\mathrm{Applicability}\right)}_{\mathrm{ij}}+\;\varepsilon_{\mathrm{ij}}$$

To examine whether the impact of parental feedback on adolescents’ mood is dependent on: (i) general self-view, (ii) perceived parental warmth or (iii) criticism, feedback valence categories were included on the first level and individual differences variables (i-iii) were included on the second level with mood as outcome:

$${\mathrm{Mood}}_{\mathrm{ij}}=\gamma_{00}+\gamma_{01}{\left(\mathrm{General}\;\mathrm{self}-\mathrm{view}\right)}_{j}+\gamma_{10}{\left(\mathrm{Negative}\right)}_{\mathrm{ij}}+\gamma_{20}{\left(\mathrm{Positive}\right)}_{\mathrm{ij}}+\upsilon_{0j}+\upsilon_{1j}{\left(\mathrm{Negative}\right)}_{\mathrm{ij}}+\upsilon_{2j}{\left(\mathrm{Positive}\right)}_{\mathrm{ij}}+\;\varepsilon_{\mathrm{ij}}$$

$${\mathrm{Mood}}_{\mathrm{ij}}=\gamma_{00}+\gamma_{01}{\left(\mathrm{Parental}\;\mathrm{warmth}\right)}_{j}+\gamma_{10}{\left(\mathrm{Negative}\right)}_{\mathrm{ij}}+\gamma_{20}{\left(\mathrm{Positive}\right)}_{\mathrm{ij}}+\upsilon_{0j}+\upsilon_{1j}{\left(\mathrm{Negative}\right)}_{\mathrm{ij}}+\upsilon_{2j}{\left(\mathrm{Positive}\right)}_{\mathrm{ij}}+\;\varepsilon_{\mathrm{ij}}$$

$${\mathrm{Mood}}_{\mathrm{ij}}=\gamma_{00}+\gamma_{01}{\left(\mathrm{Parental}\;\mathrm{criticism}\right)}_{j}+\gamma_{10}{\left(\mathrm{Nega}\mathrm{tive}\right)}_{\mathrm{ij}}+\gamma_{20}{\left(\mathrm{Positive}\right)}_{\mathrm{ij}}+\upsilon_{0j}+\upsilon_{1j}{\left(\mathrm{Negative}\right)}_{\mathrm{ij}}+\upsilon_{2j}{\left(\mathrm{Positive}\right)}_{\mathrm{ij}}+\;\varepsilon_{\mathrm{ij}}$$

#### 2.4.2 MRI data preprocessing

MRI data were pre-processed and analyzed using SPM12 (Wellcome Trust Centre for Neuroimaging, London, UK), implemented in MATLAB R2018b (MathWorks, Natick, MA). Both raw and preprocessed data were checked for quality, registration and movement (*M* = 0.09 mm, *SD* = 0.07, *range:* 0.002–3.80). All functional scans were corrected for slice-timing, corrected using field maps, unwarped and realigned, co-registered with the anatomical scan, normalized to MNI-space using the DARTEL toolbox (Ashburner, 2007), resliced to 1.5 mm^3^ voxels and spatially smoothed with an 8 mm FWHM isotropic Gaussian kernel.

#### 2.4.3 fMRI data analysis

To examine neural responses to parental feedback, we defined a general linear model (GLM) that included separate regressors for onsets of each feedback valence (i.e. 3 separate regressors for positive, intermediate and negative feedback) and an onset regressor for the mood question. Feedback onset regressors were modeled for the duration feedback was displayed on the screen (2500 ms). The mood question regressor was modeled for the duration questions were displayed on the screen (self-paced; mean duration = 1902 ms; SD = 964; range = 395–7903) and functioned as a regressor of no interest. The GLM further included six motion regressors to correct for head motion based on the realignment parameters. For each subject, t-contrasts were computed to compare positive and negative feedback to each other and to intermediate feedback.

To explore how neural responses to parental feedback varied as a function of self-rated applicability, we defined a similar GLM as described above, in which feedback onset regressors were parametrically modulated by applicability ratings. We computed t-contrasts to examine BOLD-responses to the main effect of applicability, and the interaction between feedback valence and applicability using whole-brain t-test analyses.

To explore inter-individual differences associated with: (i) adolescents’ general self-view, (ii) perceived daily parental warmth or (iii) daily parental criticism, we ran whole-brain regression analyses on the previously described contrasts with regards to valence, but without applicability, with variables (i-iii) as a between-subjects regressor.

For all whole-brain analyses, subject-specific contrast images were submitted to group level random effects analyses, which were corrected for multiple comparisons as preregistered using Family-wise Error (FWE) cluster-correction at *p* < .05 (cluster-forming threshold of *p* < .001).

## 3 Results

### 3.1 Adolescents’ affective responses to parental feedback

Adolescents rated positive feedback words (*b* = 1.05, *SE* = 0.04, *t* = 29.19, *p* < .001) as more positive than intermediate feedback words (*b* = −0.02, *SE* = 0.02, *t* = −0.90, *p* = .372, ns), which were rated as more positive than negative feedback words (*b* = −1.00, *SE* = 0.03, *t* = −29.48, *p* < .001) [χ2(2) = 1503.5, *p* < .001, Cohen’s *f*^2^ = 2.57]. Together these results clearly validate that adolescents’ valence ratings of feedback words are in line with our predetermined valence categories.

Compared to intermediate feedback (*b* = 0.05, *SE* = 0.07, *t* = 0.72, *p* = .472, ns), adolescents’ mood increased after receiving positive (*b* = 0.55, *SE* = 0.06, *t* = 9.55, *p* < .001) and decreased after receiving negative (*b* = −0.70, *SE* = 0.07, *t* = −10.4, *p* < .001) feedback from their parent [χ^2^(2) = 117.7, *p* < .001, Cohen’s *f*^2^ = 0.66].

Directly after the parental social feedback task, adolescents reported a significantly lower level of self-esteem (*b* = −0.27, *SE* = 0.09, *t* = −2.96, *p* = .004) [χ^2^(1) = 8.78, *p* = .003, Cohen’s *f*^2^ = 0.02] and relaxation (*b* = −0.18, *SE* = 0.08, *t* = −2.09, *p* = .041) [χ^2^(1) = 4.36, *p* = .037, Cohen’s *f*^2^ = 0.01], and higher level of sadness (*b* = 0.29, *SE* = 0.09, *t* = 3.05, *p* = .003) [χ^2^(2) = 9.32, *p* = .002, Cohen’s *f*^2^ = 0.02] and irritation (*b* = 0.47, *SE* = 0.12, *t* = 4.08, *p* < .001) compared to before they performed the task [χ^2^(1) = 16.7, *p* < .001, Cohen’s *f*^2^ = 0.05].

### 3.2 Adolescents’ neural responses to parental feedback

**Fig. 2:**
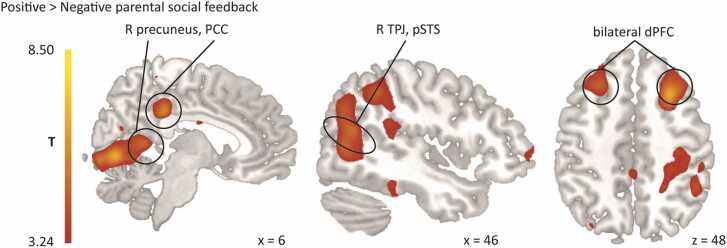
A whole-brain analysis contrasting positive with negative parental feedback resulted in activation in temporoparietal regions, dorsolateral prefrontal cortex and precuneus (thresholded at *p* < .05 using Family-wise Error (FWE) cluster-correction with a cluster-forming threshold of *p* < .001). Abbreviations: dPFC = dorsal prefrontal cortex; PCC = posterior cingulate cortex; pSTS = posterior superior temporal sulcus; TP*J* = temporoparietal junction; L = left; R = right.

On a neural level, receiving parental positive vs. negative feedback increased activity in a right PCC cluster extending into left PCC, as well as activity in right TPJ, right pSTS, and right precuneus, which were part of a large cluster with a peak in right lingual/superior temporal gyrus, extending further into right inferior parietal lobule (IPL) and right fusiform gyrus. Furthermore, we found activity in bilateral dorsal PFC (dPFC) clusters and a left angular gyrus/IPL cluster (see Fig. 2 and Table 2 for a comprehensive list of significant clusters). Compared to intermediate feedback, receiving positive parental feedback revealed no significant activations.

**Table 2:** Brain regions revealed by whole-brain regression analysis in response to positive and negative parental feedback.

*Contrast*	MNI coordinates			Voxel test value	Cluster	Cluster
Brain regions	**x**	**y**	**z**	**Z**	*p*-value	size
*Positive > Negative*						
R Lingual gyrus	14	-75	-4.5	6.82	< 0.001	15,542
	27	-60	-3	4.99		
R Superior temporal gyrus	60	-32	11	4.85		
L Calcarine fissure	-11	-93	11	6.24	< 0.001	1669
R Superior frontal gyrus (dPFC)	27	20	50	5.72	< 0.001	2000
	23	27	36	3.37		
L Middle frontal gyrus	-24	24	53	5.08	< 0.001	1318
L Superior frontal gyrus (dPFC)	-26	36	51	3.97		
L Postcentral gyrus^1^	-60	-15	24	5.02	.005	704
L Precentral gyrus	-50	-8	24	3.18		
R Posterior cingulate gyrus (PCC)	6	-35	36	4.94	< 0.001	4492
L Middle temporal gyrus	-57	-41	-15	4.80	< 0.001	1559
L Inferior temporal gyrus	-56	-33	-20	4.74		
L Middle temporal gyrus	-66	-47	-15	4.01		
L Angular gyrus^1^	-39	-68	39	4.37	< 0.001	1402
L Inferior parietal gyrus (IPL)	-50	-45	44	3.93		
R Superior frontal gyrus (dPFC)^1^	23	68	9	3.80	0.003	856
R Anterior orbital gyrus (aOFC)	41	56	-14	3.62		
R Middle frontal gyrus	47	57	2	3.48		
*Negative > Positive*						
R Anterior insula	33	23	-8	7.48	< 0.001	8433
	36	26	0	7.46		
	44	23	-5	7.40		
R Supplementary motor area	6	17	57	6.75	< 0.001	12,675
L Superior frontal gyrus, medial (dmPFC)	-5	51	24	6.61		
	0	30	48	5.23		
L Inferior frontal gyrus, triangular part	-39	26	-2	6.43	< 0.001	6775
L Anterior insula	-27	21	-14	6.01		
R Thalamus	8	-3	-2	5.21	< 0.001	2665
R Caudate nucleus (DS)	11	12	5	4.90		
L Caudate nucleus (DS)	-6	9	-2	4.71		

Notes: ^1^Cluster failed to reach significance when adding left-handedness. Neural results are corrected for multiple comparisons using Family-wise Error (FWE) cluster-correction at *p* < .05 with a cluster-forming threshold of *p* < .001. Abbreviations: aOFC = anterior orbitofrontal cortex; dmPFC = dorsomedial prefrontal cortex; dPFC = dorsal prefrontal cortex; DS = dorsal striatum; PCC = posterior cingulate cortex; L = left; R = right; MNI = Montreal Neurological Institute; Z = Z-score.

**Fig. 3:**
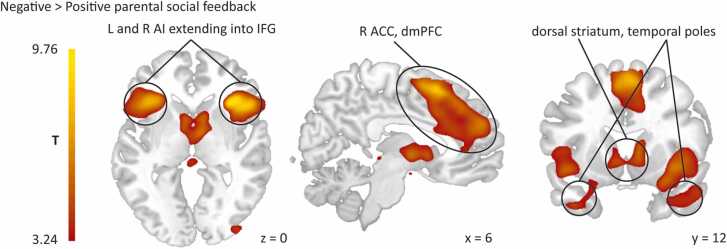
Activation in adolescents’ brain regions revealed by whole-brain regression analysis in response to negative as compared to positive parental feedback resulted in activation in anterior insula, anterior cingulate cortex, inferior frontal gyrus, and dorsomedial prefrontal cortex. Neural results are corrected for multiple comparisons using Family-wise Error (FWE) cluster-correction at *p* < .05 with a cluster-forming threshold of *p* < .001. Abbreviations: ACC = anterior cingulate cortex; AI = anterior insula; dmPFC = dorsomedial prefrontal cortex; IFG = inferior frontal gyrus; L = left; R = right.

As hypothesized, negative vs. positive parental feedback increased activity in right and left AI clusters and a bilateral dmPFC cluster extending into ACC. Both AI clusters extended into inferior frontal gyrus (IFG) and temporal pole. Furthermore, we found increased activity in a bilateral dorsal striatum (DS) cluster extending into thalamus, pallidum and VS (see Fig. 3 and Table 2 for complete list of significant clusters). Compared to intermediate feedback, receiving negative parental feedback increased activity in right and left AI/IFG clusters, see Supplementary Material 2.

### 3.3 Confound analyses

To control for age, sex, left-handedness, and length of interval between lab and MRI session, we ran additional analyses, see Supplementary Material 3. All findings remained significant when adding adolescents’ age, or length of interval as covariate. When adding left-handedness, some clusters in the positive vs. negative feedback contrast failed to reach significance, see Table 2. Additionally, adding sex as covariate revealed differences in neural processing between adolescent girls and boys, see Supplementary Material 3. Given that we had no aim or a priori hypotheses about sex differences combined with the small group sizes and skewed distribution of boys and girls, these findings should be interpreted with caution and replicated in larger samples. In exploratory analyses, we found no impact of pubertal development on affective and neural responses to parental feedback in our sample (see Table 1 and Supplementary Material 4). Finally, we explored effects of feedback from father vs. mother on mood and neural responses, indicating that altogether, adolescents seem to respond quite similar to paternal and maternal feedback (see Supplementary Material 5).

### 3.4 (In)applicability of feedback

Adolescents rated positive feedback words (*b* = 0.78, *SE* = 0.05, *t* = 15.8, *p* < .001) as more applicable to the self than intermediate words (*b* = 0.04, *SE* = 0.04, *t* = 0.90, *p* = .371, ns), and negative feedback words (*b* = −0.90, *SE* = 0.05, t = −19.7, *p* < .001) as less applicable to the self than intermediate words [χ^2^(2) = 774.9, *p* < .001, Cohen’s *f*^2^ = 0.82], illustrating that in general, adolescents have positive self-views.

**Fig. 4:**
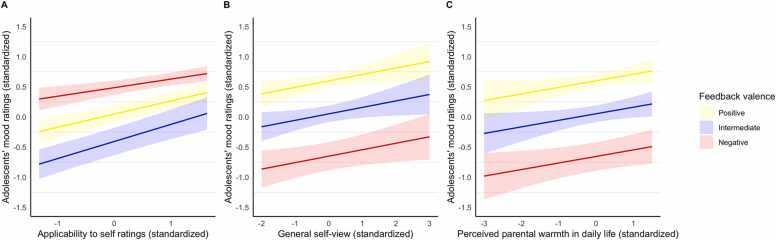
A. Interaction effect of receiving positive (yellow), intermediate (blue) and negative (red) parental, which is not (−1) or very (1) applicable (standardized) on adolescents’ mood (standardized; higher score indicates better mood), *p* = .005. B. Main effect of adolescents’ general self-view (standardized) on adolescents’ mood after receiving parental feedback (standardized), *p* = .035. C. Main effect of perceived parental warmth in daily life (standardized) on adolescents’ mood after receiving parental feedback (standardized), *p* = .036.

Adolescents’ mood decreased when feedback words were presented that were regarded as inapplicable to the self, irrespective of feedback valence [χ^2^(1) = 53.1, *p* < .001]. In addition, we found the hypothesized interaction effect between feedback valence and applicability on adolescents’ mood [χ^2^(2) = 10.4, *p* = .005, Cohen’s *f*^2^ = 0.77]. That is, adolescent mood was affected most when negative (*b* = 0.07, *SE* = 0.04, *t* = 1.68, *p* = .094, ns) and intermediate (= reference category; *b* = 0.22, *SE* = 0.03, *t* = 6.75, *p* < .001) feedback words were regarded as inapplicable, whereas mood was affected less by inapplicable positive feedback (*b* = −0.08, *SE* = 0.04, *t* = −2.13, *p* = .033), see Fig. 4A.

Whole-brain analyses testing for brain activity associated with parametric increases or decreases in applicability across feedback valence categories did not result in any significant clusters that survived correction for multiple comparisons. An analysis testing for an interaction between feedback valence and applicability did not result in significant clusters at our chosen threshold either.

### 3.5 Adolescents’ general self-view

Inter-individual differences in adolescents’ general self-view were positively associated with mood in response to parental feedback [χ^2^(1) = 4.43, *p* = .035, Cohen’s *f*^2^ = 0.70]. That is, adolescents who viewed themselves overall more positively also reported more positive mood throughout the task, independent of feedback valence (*b* = 0.11, *SE* = 0.05, *t* = 2.11, *p* = .040), see Fig. 4B. No evidence was found for our hypothesized interaction between valence and general self-view [χ^2^(2) = 3.78, *p* = .151, ns].

Whole-brain regression analyses testing for inter-individual differences in neural responses to parental feedback related to general self-view as between-subjects regressor did not yield any significant clusters that survived correction for multiple comparisons.

### 3.6 Perceived parental warmth and criticism in daily life

Adolescents reporting higher levels of perceived parental warmth in daily life reported more positive mood throughout the parental feedback task, independent of valence (*b* = 0.11, *SE* = 0.05, *t* = 2.11, *p* = .040) [χ^2^(1) = 4.41, *p* = .036, Cohen’s *f*^2^ = 0.73], see Fig. 4C. We found no evidence for our hypothesized interaction between valence and parental warmth [χ^2^(2) = 2.74, *p* = .254, ns]. We furthermore found no evidence for impact of perceived parental criticism on mood responses to parental feedback [main effect: χ^2^(1) = 0.27, *p* = .604, ns]; [interaction effect: χ^2^(2) = 2.39, *p* = .303, ns].

Whole-brain analyses testing for inter-individual differences in brain responses to parental feedback using parental warmth or criticism scores as between-subjects regressors did not result in significant clusters at our chosen threshold.

### 3.7 Associations between general self-view and perceived parental warmth and criticism

To test the robustness of our findings related to general self-view and perceived parental warmth and criticism, we created a model predicting adolescents’ mood including all three inter-individual variables:

$${\mathrm{Mood}}_{\mathrm{ij}}=\gamma_{00}+\gamma_{01}{\left(\mathrm{General}\;\mathrm{self}-\mathrm{view}\right)}_{j}+\gamma_{02}{\left(\mathrm{Parental}\;\mathrm{warmth}\right)}_{j}+\gamma_{03}{\left(\mathrm{Parental}\;\mathrm{criticism}\right)}_{j}+\gamma_{10}{\left(\mathrm{Negative}\right)}_{\mathrm{ij}}+\gamma_{20}{\left(\mathrm{Positive}\right)}_{\mathrm{ij}}+\upsilon_{0j}+\upsilon_{1j}{\left(\mathrm{Negative}\right)}_{\mathrm{ij}}+\upsilon_{2j}{\left(\mathrm{Positive}\right)}_{\mathrm{ij}}+\;\varepsilon_{\mathrm{ij}}$$

In this model, the main effect of perceived parental warmth remained a significant predictor of mood (*b* = 0.13, *SE* = 0.06, *t* = 2.32, *p* = .024) [χ2(1) = 5.38, *p* = .020, Cohen’s *f*^2^ = 0.73], but the main effect of general self-view was no longer fully significant (*b* = 0.09, *SE* = 0.05, *t* = 1.71, *p* = .093, ns) [χ2(1) = 2.92, *p* = .087, ns]. Given the correlation between general self-view and perceived parental warmth (*r*(55) = 0.31, *p* = .020), these findings indicate that perceived parental warmth may be the driving force in predicting adolescents’ mood after parental feedback. However, replication in larger samples could shed light on the robustness of these associations.

## 4 Discussion

This study investigated adolescents’ affective and neural responses to social feedback supposedly given by their parent. We examined these responses in relation to both intrapersonal (i.e., consistency with self-views and one’s general self-view) and interpersonal factors (i.e., daily life parental warmth and criticism). Our results show, as expected, that positive feedback from a parent increases adolescent mood and that negative feedback decreases it. On a neural level, positive parental feedback was associated with increased activity in brain regions supporting social cognition, including TPJ, pSTS, and precuneus/PCC. Negative parental feedback was associated with increased activity in areas related to salience processing (i.e., AI, ACC, and DS) and social cognition (i.e., dmPFC, IFG, and temporal poles). Our analyses demonstrated that when parental feedback did not match adolescents’ views of themselves, their mood decreased, especially when negative feedback was not in line with their self-views. In terms of individual differences, adolescents with a relatively more positive self-view and high levels of perceived parental warmth in daily life reported higher mood throughout the task. However, we found no convincing evidence that these individual differences impacted neural responses to parental feedback.

As hypothesized, parental criticism was related to increased activation in neural areas relevant for saliency, i.e. ACC, AI, and DS, consistent with neural responses that are generally found in the context of negative social feedback from strangers (Cacioppo et al., 2013, Eisenberger et al., 2011, Feng et al., 2021, Fritz et al., 2020, Kawamichi et al., 2018, Muscatell et al., 2016, Rotge et al., 2015, Schindler et al., 2019, van Schie et al., 2018, Will et al., 2016). Interestingly, in line with van Schie et al. (2018), using a similar task, we found increased ACC activity specifically in the anterior MCC and pgACC, sub-regions particularly related to social pain and negative feelings related to social pain (Rotge et al., 2015). Moreover, also activity in VS was found in response to negative vs. positive parental feedback. VS activity in response to negative social experiences (e.g. social exclusion) is more often reported in adolescents, but not in young adults (Vijayakumar et al., 2017). This suggests that feelings in response to *negative* feedback are particularly salient for adolescents (Lamblin et al., 2017) and dovetails with prior studies proposing that adolescents, relative to adults, may internalize negative feedback to a greater extent, leaving them more vulnerable to social feedback (Rappaport and Barch, 2020, Rodman et al., 2017, Yoon et al., 2018). It also aligns with the notion that adolescents reported lower self-esteem and more negative feelings after finishing our task, despite receiving an equivalent number of words related to parental praise vs. criticism. This is in contrast with the adult sample of van Schie et al. (2018), where self-esteem was not lowered after the task, suggesting that adolescents may have more difficulties in recovering from (parental) negative feedback. Thus, even in this group of healthy adolescents with generally positive parent-child bonds, parental criticism is emotionally salient and negatively impacts their self-esteem.

Notably, parental praise did not increase activity in VS and vmPFC, as was expected based on prior studies involving positive feedback (Davey et al., 2010, Feng et al., 2021, Gunther Moor et al., 2010, Izuma et al., 2008, Kawamichi et al., 2018, Korn et al., 2012, Morelli et al., 2015, Muscatell et al., 2016, Schindler et al., 2019, Will et al., 2020, Will et al., 2017). However, we did find increased activity in right TPJ, right pSTS, right precuneus, and PCC in response to parental praise. These activated brain regions noticeably overlap with the ‘default mode network’, including the PCC, TPJ, IPL, and precuneus, which is robustly found to be activated in studies using resting-state functional connectivity, a widely used technique to investigate neural processing at rest (Greicius et al., 2003). It has been argued that this default activity may reflect the representation of the self, the so-called ‘default self’ (Qin and Northoff, 2011, Yang et al., 2016). This might suggest that in typically developing adolescents – for whom receiving parental praise (which is largely in line with their own self-views) may be a relatively common experience – processing this praise may rely on more internal default state activity of the brain, and hence may not result in increased reward-related activity. Likewise, receiving parental criticism may be less common and/or less expected, which might explain the increased activation related to saliency.

Both parental praise and criticism increased activity in socio-cognitive related regions (TPJ, pSTS, precuneus, PCC and dmPFC, IFG, temporal poles respectively), which dovetails with prior work examining neural responses to feedback from unfamiliar people (Kawamichi et al., 2018, Molenberghs et al., 2016, Muscatell et al., 2016, van Houtum et al., 2021, van Schie et al., 2018). The elicited socio-cognitive processes might be crucial for adequately dealing with parental feedback, and reflecting on their parent’s intentions underlying feedback. Our paradigm, using higher-order personality feedback from one’s parent (i.e., someone who knows you extremely well), seems to elicit more socio-cognitive processing than typical social evaluation studies using more ‘basic reinforcers’, such as receiving likes or being excluded during the Cyberball game. Remarkably, parental praise and criticism activate different components of the socio-cognitive network, consistent with previous research reporting that ‘feeling understood’ – which may represent more interpersonal closeness – activated TPJ and precuneus, whereas ‘not feeling understood’ activated dmPFC (Morelli et al., 2014). However, further research is needed to better understand the delineated patterns of activity in socio-cognitive areas in response to positive and negative feedback.

We furthermore found that *in*consistency of parental feedback with adolescents’ pre-existing beliefs about themselves resulted in decreased mood. Especially parental criticism regarded as inapplicable impacted mood negatively, whereas this was less the case for inapplicable parental praise. These findings are strikingly similar to prior research investigating the impact of applicability of social feedback on mood from unfamiliar persons in adults (van Schie et al., 2018), and vicarious feedback about one’s own child (van Houtum et al., 2021). According to the self-verification model, people are motivated to seek information that confirms their self-views, even when these are negative (Vandellen et al., 2011). In that sense, self-views may work as a ‘filter’ through which feedback is received. However, we found no impact of applicability of parental feedback on adolescents’ neural responses, whereas van Schie et al. (2018) found increased precuneus activation in adults. In contrast, prior self-evaluation studies in adolescents found valence-dependent recruitment of vmPFC and pgACC in response to endorsement of personality characteristics (Barendse et al., 2020, Cosme et al., 2019), which would imply an interaction effect between applicability and feedback valence. Replication studies and larger sample sizes are needed to draw valid conclusions on the impact of applicability of parental feedback on a neural level.

In terms of individual differences, adolescents with a more positive self-view and increased perceived parental warmth in daily life reported more positive mood throughout the task, regardless of the valence of the feedback received. Having a more positive self-view may translate into a more stable self-image as well as a higher motivation to maintain one’s positive self-view, which might explain why these adolescents have an overall higher mood level after parental feedback (Alicke et al., 2020, Harter, 2015, Taylor and Brown, 1988, Vandellen et al., 2011). Notably, a more positive self-view was not associated with the VAS-ratings before and after the task, suggesting that these findings are specific for the immediate reactions to parental feedback. It should be noted though that given the association between general self-view and perceived parental warmth, it may be premature to draw strong conclusions. The findings related to parental warmth are in line with the parental acceptance-rejection theory, as adolescents perceiving *less* parental warmth tend to experience interpersonal contexts as more negative (Butterfield et al., 2020, Rohner et al., 2005). Although it is plausible that frequency and/or intensity of parental criticism on a daily basis may impact how adolescents emotionally react to parental feedback, no associations were found in our sample, possible due to the low levels of reported criticism. On a neural level, we found no evidence for these individual differences impacting brain responses to parental feedback differently. Given that our sample rated themselves quite positively and experienced their parent as quite warm and not very critical, it would be interesting to look at (sub)clinical or at-risk samples, e.g. adolescents with depression, where a negative self-image is prominent (Beck and Alford, 2009, van Schie et al., 2018, Will et al., 2020), and a broader range of parental warmth and criticism is often reported (Pinquart, 2017, Restifo and Bögels, 2009, Yap et al., 2014).

Our study had several strengths. First, we employed an ecologically valid paradigm, using realistic social feedback purportedly from one’s own parent, with a credible cover story and a sensitive debriefing method. Parents are likely to have more information to make appropriate appraisals about their child’s personality characteristics, making them a more accurate feedback source as compared to unknown or less familiar others (Bollich et al., 2011, Silva et al., 2020, Vazire, 2010). Additionally, parents may be particularly influential in shaping and adapting self-views across adolescence (Carmichael et al., 2007, Silva et al., 2020). Moreover, this study not only examined valence of parental feedback, including both mothers and fathers, it also incorporated the impact of self-views (both per feedback word and general self-view) and daily life parenting perceptions. Finally, by examining neural responses to parental praise in adolescence this study contributes to the field, as work on normative development of neural responses to positive feedback is still sparse (Rappaport and Barch, 2020).

Our study also had some limitations. First, adolescents did not re-evaluate the applicability of personality characteristics, which could have given insights in updating processes of one’s self-views after receiving parental feedback. Previous research showed that adolescents, compared to adults, updated their self-views more negatively after receiving negative feedback from peers, possibly indicating that self-protecting biases emerge later in development (Rodman et al., 2017). Furthermore, as we only incorporated feedback from parents in our design, we were not able to investigate whether parental feedback is differentially impactful compared to feedback from less significant others. With respect to the EMA measures of parenting in daily life, adolescents reported on parental warmth and criticism by asking *‘how warm/loving and critical the parent was towards the adolescent throughout the day’*. Despite the fact that considerable variation was reported on these questions throughout the 14 days, indicating that adolescents reported on specific parenting behaviors, it should be noted that this may also in part reflect adolescents’ *general* positive or negative perceptions of their parent. Finally, we did not ask adolescents to what extent the personality characteristics would apply to them according to their parent, i.e. reflected self-evaluations, which are potentially internalized in one’s self-concept (Silva et al., 2020, Van der Cruijsen et al., 2019), and may accordingly moderate mood and neural responses to parental feedback. Probably, expected parental feedback – even though inconsistent with own self-views – is less surprising and painful (or rewarding) as compared to unexpected feedback.

### 4.1 Conclusions

Our findings augment prior work by demonstrating that adolescents – depending on both intra- and interpersonal factors – are emotionally affected by *parental* social feedback. Especially receiving inapplicable parental criticism has a negative impact on adolescents’ mood. Whereas receiving both parental praise and criticism engage socio-cognitive related brain regions, parental criticism additionally activates areas important for social saliency. Together with the notion that adolescents also reported more negative feelings after receiving parental feedback, despite an equal mix of positive and negative feedback, our results may imply that particularly *negative* parental feedback is emotionally salient to adolescents. Moreover, as internalizing disorders typically develop during adolescence (Costello et al., 2011), in which particularly (self-)negativity bias, rejection sensitivity and low self-esteem are often central components (Rappaport and Barch, 2020), future studies should examine how individual variations in self-views and parental behaviors relate to these neural responses, well-being and mental health in adolescence. Our insights may also have clinical implications, as awareness of both adolescents’ own self-views and reactions to parental feedback, as well as parental awareness of the potential effects of giving feedback (both praise and criticism), might be key targets in parent-adolescent communication interventions and strategies for adolescent internalizing disorders. The current study may have laid a first foundation for investigating (neural) underlying mechanisms related to these clinical aspects.

## Funding

This work was supported by the 10.13039/501100003246Netherlands Organisation for Scientific Research (NWO), The Netherlands [VICI Grant number 453-15-006 to B.M.E.]. G-J.W. was funded by Marie Skłodowska-Curie Grant agreement No. 707404, European Union, and Sara van Dam z.l. Foundation, 10.13039/501100001722Royal Netherlands Academy of Arts and Sciences, the Netherlands. Apart from financial contribution, these funding agencies had no role in the study design, collection, analysis, and data interpretation or in writing the manuscript.

## CRediT authorship contribution statement

**Lisanne A.E.M. van Houtum**: Conceptualization, Methodology, Software, Investigation, Formal analysis, Writing – original draft, Visualization. **Geert-Jan Will**: Conceptualization, Methodology, Software, Investigation, Writing – review & editing, Supervision. **Mirjam C.M. Wever**: Conceptualization, Software, Investigation. **Loes H.C. Janssen**: Investigation. **Charlotte C. van Schie**: Conceptualization, Methodology, Software, Writing – review & editing, Supervision. **Marieke S. Tollenaar**: Conceptualization, Writing – review & editing, Supervision. **Bernet M. Elzinga**: Conceptualization, Writing – review & editing, Supervision, Funding acquisition.

## Declaration of Competing Interest

The authors declare that they have no known competing financial interests or personal relationships that could have appeared to influence the work reported in this paper.

## Data Availability

The de-identified data, analysis scripts and materials for this study are available on DataverseNL and the MRI data are available on Neurovault (https://neurovault.org/collections/10384/). For any questions or additional material, please contact the corresponding author.
