# Supplementary material for: Adolescents’ affective and neural responses to parental praise and criticism
Source: Dev Cogn Neurosci. 2022 Mar 15;54:101099. doi: 10.1016/j.dcn.2022.101099 (PMC8933824; doi:10.1016/j.dcn.2022.101099)
Supplement: Supplementary file 1 — Supplementary material [file mmc1.docx]

**Supplementary Material**

1. ***Manipulation check interview***

Upon completion of scanning, we conducted a manipulation check interview to assess whether participants believed that their parents actually provided the feedback they received in the scanner. This interview was audio recorded, see Supplementary Table S1 for the specific questions.

To assess doubts about the authenticity of feedback, we used a funneling suspicion probe derived from prior feedback studies (van Houtum et al., 2021; van Schie et al., 2018). The goal of this probe was to first ask three general questions about the task (i.e. questions 1-3) to allow those who had strong disbeliefs about the task to express their disbeliefs and/or doubts spontaneously. The second part of the interview consisted of three additional questions becoming more and more explicit one by one about potential deception and questioning the authenticity of feedback (i.e. questions 4-6), to assess more subtle indications of doubt. We categorized participants in three groups: i) ‘non-believers’ (i.e., those who showed spontaneous expressions of disbelief during questions 1-3), ii) ‘mild doubters’ (i.e., those who expressed some doubt in response to questions 1-6, but no serious disbelief about the task), and iii) 'full-believers’ (i.e, those who showed no expressions of doubt, not even to questions 4-6 implicitly mentioning deception). Three research assistants independently judged whether each participant should be assigned to the full-believer, mild doubter, or non-believer category, by listening to the recorded interviews. In case of inter-rater disagreement, the final rater (LvH) made a final categorization decision (disagreements across raters: *n* = 18; 30.5%).

Adolescents either believed the cover story completely (full-believers: *n* = 18/59, 30.5%) or expressed some doubt (mild doubters: *n* = 41/59, 69.5%), while no one could be categorized as non-believer (*n* = 0/59, 0%) (see *Methods* section in main text). For example, mild doubters expressed in response to questions 1-3: *‘I doubted at a certain point, like huh, did my mother really say that?’*, or in response to question 4: *‘I already was not sure whether you made this up or not’,* while full-believers e.g. responded to question 4 with: *‘I am very sure that my parent gave the feedback’.*

Additionally, we explored whether full-believers and mild doubters showed differences in affective responses to parental feedback. Predetermined valence categories (i.e. positive, intermediate (= reference category), and negative feedback) were specified on the first level and ‘belief status’ (full-believer vs. minor doubter) was included on the second level, with adolescents’ mood after each feedback word as outcome:

$${Mood}_{ij}=\gamma_{00}+\gamma_{01}{(Belief status)}_{j}+\gamma_{10}{(Negative)}_{ij}+\gamma_{20}{(Positive)}_{ij}+\gamma_{11}{(Belief status)}_{j}{(Negative)}_{ij}+\gamma_{21}{(Belief status)}_{j}{(Positive)}_{ij}+\upsilon_{0j}+\upsilon_{1j}{(Negative)}_{ij}+\upsilon_{2j}{(Positive)}_{ij}+ \varepsilon_{ij}$$

We found no main effect of belief status on adolescents’ mood after receiving parental feedback was found (*b* = 0.21, *SE* = 0.15, *t* = 1.38, *p* = .172, ns) [χ2(1) = 0.25, *p* = .615, ns], neither an interaction effect between feedback valence and belief status [χ2(2) = 2.51, *p* = .285, ns].”

**Supplementary Table S1.** Verbally asked questions during manipulation check interview of parental social feedback task in Dutch (left) and English (right)

| *Question* | |  | |  |
| --- | --- | --- | --- | --- |
| 1 | In hoeverre ben je het eens met de feedbackwoorden die je ontvangen hebt van je ouder? | | To what extent do you agree with the feedback words you have received from your parent? | |
| 2 | Hoe denk je nu over je vader/moeder na het krijgen van de feedback? | | How do you feel about your father/mother after getting the feedback? | |
| 3 | Heeft de feedback je emotioneel geraakt? Waarom wel of niet? | | Were you emotionally affected by the feedback? Why (not)? | |
| 4 | Hoe zeker ben je ervan dat je ouder de feedback heeft gegeven? | | How confident are you that your parent gave the feedback? | |
| 5 | Dacht je dat de hele tijd? Vanaf wanneer wel of niet? | | Did you have this feeling during the whole task? Or at what point did this feeling change? | |
| 6 | Wat waren redenen om te twijfelen aan de opzet? | | What were reasons to doubt the task setup? | |

1. ***Neural findings in response to negative vs. intermediate parental feedback***

**Supplementary Table S2.** Brain regions revealed by whole-brain regression analysis in response to negative vs. intermediate parental feedback

| *Contrast* | MNI coordinates | | | Voxel test value | Cluster | Cluster |
| --- | --- | --- | --- | --- | --- | --- |
| Brain regions | **x** | **y** | **z** | **Z** | ***p*-value** | **size** |
| *Negative > Intermediate* |  |  |  |  |  |  |
| R Anterior insula | 27 | 20 | -15 | 5.26 | <.001 | 1937 |
| R Inferior frontal gyrus, orbital part | 32 | 26 | -8 | 4.57 |  |  |
| R Anterior insula | 44 | 24 | -3 | 4.37 |  |  |
| L Posterior orbital gyrus (pOFC) | -32 | 23 | -14 | 4.61 | .003 | 887 |
| L Inferior frontal gyrus, triangular part | -36 | 29 | 0 | 3.95 |  |  |

Notes: Neural results are corrected for multiple comparisons using Family-wise Error (FWE) cluster-correction at *p* < .05 with a cluster-forming threshold of *p* < .001. Abbreviations: pOFC = posterior orbitofrontal cortex; L = left; R = right; MNI = Montreal Neurological Institute; Z = Z-score.

**Supplementary Figure 1.** Activation in adolescents’ brain regions revealed by whole-brain regression analysis in response to in response to negative vs. intermediate parental feedback in anterior insula and inferior frontal gyrus. Neural results are corrected for multiple comparisons using Family-wise Error (FWE) cluster-correction at *p* < .05 with a cluster-forming threshold of *p* < .001. Abbreviations: AI = anterior insula; IFG = inferior frontal gyrus; L = left; R = right.

1. ***Confounds and exploration of sex differences***

Results from analyses on behavioral measures did not change when adding sex, age, parental sex or length of interval between lab and MRI session as covariate. Regarding the neural results, only minor changes in location of peak coordinates were observed when taking age or length of interval into account in the neural analyses, and some clusters failed to reach significance at our chosen threshold when adding left-handedness as a covariate (see *Results* section and Table 2 in main text for relevant clusters).

When adding sex as a covariate, this resulted in different neural findings in the sample of girls (*n* = 39) vs. the sample of boys (*n* = 20). Overall, girls exhibited larger clusters and more cluster activation in response to parental feedback compared to boys. This was more pronounced in response to positive parental feedback (vs. negative feedback; see Supplementary Table S3). We remain agnostic to potential causes of differential findings in boys and girls in our sample, but it should be noted that the sample of boys was too small to draw reliable conclusions from activation patterns across boys. Specifically, when receiving positive vs. negative parental feedback, for girls all previously reported clusters remained significant except for the left post/precentral gyrus, left dPFC, left angular gyrus/IPL, and right aOFC clusters, whereas for boys only a small left lingual gyrus cluster (i.e. *not* extending into right TPJ, right pSTS, right IPL, right precuneus, and right fusiform gyrus) remained significant. See Supplementary Table S3 for complete overview of all significant clusters.

On the other hand, when receiving negative vs. positive parental feedback, all clusters remained significant in girls. However, in boys, activity in right dorsal striatum, right thalamus, right pallidum, and right VS failed to reach significance. See Supplementary Table S3 for complete overview of significant clusters. For both girls and boys, no significant cluster-activation was found when receiving negative vs. intermediate parental feedback.

**Supplementary Table S3.** Brain regions revealed by whole-brain regression analysis in response to positive and negative parental feedback with adolescents’ sex added as covariate (0 = girls, 1 = boys)

| *Contrast* | MNI coordinates | | | Voxel test value | Cluster | Cluster |
| --- | --- | --- | --- | --- | --- | --- |
| Brain regions | **x** | **y** | **z** | **Z** | ***p*-value** | **size** |
| *Positive > Negative* |  |  |  |  |  |  |
| R Lingual gyrus | 14 | -77 | -8 | 5.89 | <.001 | 12033 |
| R Angular gyrus | 36 | -66 | 41 | 5.04 |  |  |
| R Lingual gyrus | 23 | -69 | -3 | 5.03 |  |  |
| L Calcarine fissure | -11 | -93 | 12 | 5.58 | <.001 | 1291 |
|  | -8 | -95 | 3 | 5.14 |  |  |
| R Superior frontal gyrus (dPFC) | 27 | 18 | 47 | 4.89 | .001 | 1065 |
| L Inferior temporal gyrus | -56 | -41 | -17 | 4.41 | .001 | 1000 |
|  | -53 | -35 | -21 | 4.17 |  |  |
| L Middle temporal gyrus | -69 | -41 | -12 | 3.86 |  |  |
| L Middle occipital gyrus | -36 | -71 | 41 | 4.14 | .001 | 1000 |
| *Negative > Positive* |  |  |  |  |  |  |
| R Anterior insula | 35 | 26 | 0 | 6.28 | <.001 | 4942 |
|  | 32 | 18 | -15 | 6.06 |  |  |
|  | 44 | 23 | -5 | 5.78 |  |  |
| R Supplementary motor area | 6 | 18 | 57 | 5.90 | <.001 | 8777 |
| L Superior frontal gyrus, medial (dmPFC) | -6 | 51 | 26 | 5.89 |  |  |
| R Supplementary motor area | 6 | 5 | 63 | 5.03 |  |  |
| R Middle temporal gyrus | 47 | 3 | -26 | 5.17 | .003 | 863 |
| R Inferior temporal gyrus | 45 | -5 | -36 | 3.88 |  |  |
| R Middle temporal pole | 36 | 11 | -35 | 3.36 |  |  |
| L Inferior frontal gyrus, triangular part | -39 | 26 | -2 | 4.99 | <.001 | 2710 |
| L Anterior insula | -27 | 20 | -14 | 4.97 |  |  |
| L Inferior frontal gyrus, triangular part | -50 | 20 | 5 | 4.04 |  |  |
| R Caudate nucleus (DS) | 9 | 9 | 2 | 5.44 | <.001 | 1153 |
| R Thalamus | 8 | -3 | -2 | 4.87 |  |  |
| L Caudate nucleus (DS) | -5 | 9 | -2 | 4.05 |  |  |

Notes: Neural results are corrected for multiple comparisons using Family-wise Error (FWE) cluster-correction at *p* < 0.05 with a cluster-forming threshold of *p* < 0.001. Abbreviations: dPFC = dorsal prefrontal cortex; dmPFC = dorsomedial prefrontal cortex; DS = dorsal striatum; L = left; R = right; MNI = Montreal Neurological Institute; Z = Z-score.

Due to these differential activation patterns related to sex, we further explored *actual* sex differences by directly contrasting girls against boys. First, we explored whether girls and boys differed in their affective responses to parental feedback. Predetermined valence categories (i.e. positive, intermediate, and negative feedback) were specified on the first level and sex was included on the second level, with adolescents’ mood after each feedback word as outcome. No main effect of sex on adolescents’ mood after receiving parental feedback was found (*b* = -0.26, *SE* = 0.15, *t* = -1.73, *p* = .088, ns) [χ2(1) = 0.99, *p* = .319, ns], nor an interaction effect between valence and sex [χ2(2) = 3.21, *p* = .201, ns].

Next, we explored sex differences within neural responses by using a two-sample t-test analysis on the second level. When comparing girls against boys when receiving negative vs. intermediate parental feedback, girls exhibited increased activation in a left dorsolateral PFC (dlPFC) cluster extending into IFG. No other differences were observed; see Supplementary Table S4.

**Supplementary Table S4.** Brain regions revealed by exploratory whole-brain regression analysis in response to negative vs. intermediate parental feedback when comparing girls against boys

| *Contrast* | MNI coordinates | | | Voxel test value | Cluster | Cluster |
| --- | --- | --- | --- | --- | --- | --- |
| Brain regions | **x** | **y** | **z** | **Z** | ***p*-value** | **size** |
| *Negative > Intermediate* |  |  |  |  |  |  |
| L Middle frontal gyrus (dlPFC) | -38 | 57 | 12 | 4.18 | .001 | 1036 |
|  | -47 | 48 | 0 | 3.82 |  |  |
|  | -41 | 54 | 21 | 3.52 |  |  |

Notes: Neural results are corrected for multiple comparisons using Family-wise Error (FWE) cluster-correction at *p* < 0.05 with a cluster-forming threshold of *p* < 0.001. Abbreviations: dlPFC = dorsolateral prefrontal cortex; L = left; MNI = Montreal Neurological Institute; Z = Z-score.

***4. Exploration of impact of pubertal development on affective and neural responses***

We additionally explored whether pubertal development was related to affective and neural responses to parental feedback. We assessed the Pubertal Development Scale (PDS; Peterson et al., 1988), a self-report scale about development of secondary sexual characteristics (see Braams et al., 2015 for similar assessment). First, we explored effects related to affective responses. Predetermined valence categories (i.e. positive, intermediate (= reference category), and negative feedback) were specified on the first level and level of pubertal development was included on the second level, with adolescents’ mood after each feedback word as outcome:

$${Mood}_{ij}=\gamma_{00}+\gamma_{01}{(Pubertal development)}_{j}+\gamma_{10}{(Negative)}_{ij}+\gamma_{20}{(Positive)}_{ij}+\gamma_{11}{(Pubertal development)}_{j}{(Negative)}_{ij}+\gamma_{21}{(Pubertal development)}_{j}{(Positive)}_{ij}+\upsilon_{0j}+\upsilon_{1j}{(Negative)}_{ij}+\upsilon_{2j}{(Positive)}_{ij}+ \varepsilon_{ij}$$

No main effect of pubertal development on adolescents’ mood after receiving parental feedback was found (*b* = 0.00, *SE* = 0.07, *t* = 0.01, *p* = .993, ns) [χ2(1) = 0.15, *p* = .697, ns], nor an interaction effect between feedback valence and pubertal development [χ2(2) = 1.52, *p* = .468, ns].

On the neural level, whole-brain regression analyses testing for inter-individual differences in neural responses to parental feedback related to pubertal development as between-subjects regressor did not yield in any significant clusters that survived correction for multiple comparisons.

Taken together, these analyses show no evidence of an impact of pubertal development on affective and neural responses to parental feedback in our sample.

1. ***Exploration of parental sex differences***

We explored parental sex differences by directly contrasting adolescents receiving feedback from their father (*n* = 27; 16 girls, 11 boys) as compared to adolescents receiving feedback from their mother (*n* = 32; 23 girls, 9 boys). First, we explored differences in affective responses to feedback from father vs. mother. Predetermined valence categories (i.e. positive, intermediate (= reference category), and negative feedback) were specified on the first level and parental sex was included on the second level, with adolescents’ mood after each feedback word as outcome:

$${Mood}_{ij}=\gamma_{00}+\gamma_{01}{(Parental sex)}_{j}+\gamma_{10}{(Negative)}_{ij}+\gamma_{20}{(Positive)}_{ij}+\gamma_{11}{(Parental sex)}_{j}{(Negative)}_{ij}+\gamma_{21}{(Parental sex)}_{j}{(Positive)}_{ij}+\upsilon_{0j}+\upsilon_{1j}{(Negative)}_{ij}+\upsilon_{2j}{(Positive)}_{ij}+ \varepsilon_{ij}$$

We did not find a main effect of parental sex on adolescents’ mood after receiving parental feedback (*b* = 0.12, *SE* = 0.14, *t* = 0.82, *p* = .418, ns) [χ2(1) = 2.93, *p* = .087, ns], neither an interaction effect between feedback valence and parental sex [χ2(2) = 0.58, *p* = .747, ns].

Next, we explored differences within neural responses when receiving feedback from father vs. mother by using a two-sample t-test analysis on the second level. When comparing adolescents receiving positive vs. intermediate parental feedback from father vs. mother, receiving feedback from father resulted in increased activation in a right IPL/angular gyrus cluster extending into dorsal TPJ, see Supplementary Table S6. However, when contrasting adolescents receiving positive vs. negative feedback no differences in neural processing were observed. This was also the case for negative vs. positive feedback, and negative vs. intermediate feedback from father vs. mother. So, altogether, adolescents do seem to respond quite similar to feedback from fathers vs. those of mothers.

**Supplementary Table S6.** Brain regions revealed by exploratory whole-brain regression analysis in response to positive vs. intermediate feedback from father as compared to mother.

| *Contrast* | MNI coordinates | | | Voxel test value | Cluster | Cluster |
| --- | --- | --- | --- | --- | --- | --- |
| Brain regions | **x** | **y** | **z** | **Z** | ***p*-value** | **size** |
| *Positive > Intermediate* |  |  |  |  |  |  |
| R Inferior parietal gyrus (IPL) | 57 | -51 | 44 | 4.64 | <.001 | 1421 |
| R Angular gyrus | 48 | -54 | 32 | 3.59 |  |  |

Notes: Neural results are corrected for multiple comparisons using Family-wise Error (FWE) cluster-correction at *p* < 0.05 with a cluster-forming threshold of *p* < 0.001. Abbreviations: IPL = inferior parietal lobe; L = left; MNI = Montreal Neurological Institute; Z = Z-score.

**References**

Braams, B. R., van Duijvenvoorde, A. C., Peper, J. S., & Crone, E. A. (2015). Longitudinal changes in adolescent risk-taking: a comprehensive study of neural responses to rewards, pubertal development, and risk-taking behavior. *Journal of Neuroscience*, *35*(18), 7226-7238.

Petersen, A. C., Crockett, L., Richards, M., & Boxer, A. (1988). A self-report measure of pubertal status: Reliability, validity, and initial norms. *Journal of Youth and Adolescence*, *17*(2), 117-133.

van Houtum, L. A., Wever, M., Janssen, L. H., van Schie, C. C., Will, G.-J., Tollenaar, M. S., & Elzinga, B. M. (2021). Vicarious Praise and Pain: Parental Neural Responses to Social Feedback about Their Adolescent Child. *Social Cognitive and Affective Neuroscience*.

van Schie, C. C., Chiu, C. D., Rombouts, S., Heiser, W. J., & Elzinga, B. M. (2018). When compliments do not hit but critiques do: an fMRI study into self-esteem and self-knowledge in processing social feedback. *Soc Cogn Affect Neurosci*, *13*(4), 404-417. <https://doi.org/10.1093/scan/nsy014>
